# Simplified Vocal Efficiency Metrics Normalize Following Voice Therapy in Subgroups of Patients With Nonphonotraumatic Vocal Hyperfunction

**DOI:** 10.1044/2025_AJSLP-25-00040

**Published:** 2025-08-13

**Authors:** Zilan Zhu, Jarrad H. Van Stan, Hamzeh Ghasemzadeh, Ahsan J. Cheema, Jeremy Wolfberg, Robert E. Hillman, Annie B. Fox, Daryush D. Mehta

**Affiliations:** a MGH Institute of Health Professions, Boston, MA; b Center for Laryngeal Surgery and Voice Rehabilitation, Massachusetts General Hospital, Boston; c Department of Surgery, Harvard Medical School, Boston, MA; d Speech and Hearing Bioscience and Technology, Division of Medical Sciences, Harvard Medical School, Boston, MA

## Abstract

**Purpose:**

The purpose of this study is to determine whether simplified vocal efficiency (VE) metrics could accurately identify changes after voice therapy in individuals with nonphonotraumatic vocal hyperfunction (NPVH). This study analyzes treatment-related changes for traditional VE—vocal intensity (square of sound pressure) over aerodynamic power—and three simplified VE ratios: (a) sound pressure level over aerodynamic power (SPL/AP), (b) SPL over subglottal pressure (SPL/Ps), and (c) SPL over airflow (SPL/AFLOW).

**Method:**

Retrospective data from 108 adults (80 females, 28 males) diagnosed with primary muscle tension dysphonia (associated with NPVH) and 208 vocally healthy adults (181 females, 27 males). Study participants produced repeated consonant–vowel utterances in comfortable and loud conditions before and after voice therapy, with acoustic SPL and aerodynamic measurements (Ps and AFLOW) derived. Pre- to posttherapy VE changes were analyzed using mixed-design analysis of variance models. In an exploratory analysis, patients were divided into three subgroups based on their pretherapy VE measures to investigate treatment effects within NPVH subgroups.

**Results:**

Pre- to posttherapy VE changes were not observed for the NPVH group as a whole. A subsequent subgroup analysis revealed treatment effects within female patients with NPVH exhibiting lower and higher than typical pretherapy VE metrics. SPL/Ps exhibited a treatment effect in both loudness conditions and migration toward normative ranges following therapy. Posttherapy changes were observed to varying degrees in both loudness conditions for traditional VE and simplified VE metrics of SPL/AP and SPL/AFLOW.

**Conclusions:**

VE ratios, especially SPL/Ps, demonstrate potential as metrics for evaluating the outcome of voice therapy in individuals with NPVH and aid in stratifying individuals with NPVH into subgroups compared to vocally healthy values. Further investigations are warranted to investigate the role of VE metrics in the assessment, treatment, and prevention of NPVH.

Nonphonotraumatic vocal hyperfunction (NPVH) is a behavior associated with a class of voice disorders that affect approximately 10% of the U.S. population (Dworkin-Valenti et al., 2018). NPVH is characterized by excessive activity of perilaryngeal muscles during phonation in the absence of vocal fold lesions (Oates & Winkworth, 2008). Symptoms related to NPVH include vocal strain, roughness, and breathiness (Jani et al., 2008), with several formal diagnoses including primary muscle tension dysphonia (Desjardins et al., 2022; Hillman et al., 2020) and functional dysphonia (Roy, 2003). Individuals with NPVH can experience a significant reduction in their quality of life due to communication difficulties, social isolation, employment-related difficulties, and psychological distress (Wilson et al., 2002).

This investigation studies individuals who underwent treatment for NPVH. Voice therapy is the most commonly used approach to treat NPVH, with the primary goal being to minimize voice symptoms through manipulating phonation, breathing, articulation, laryngeal posture, and so forth (Jani et al., 2008). Currently, evaluating the effects of treatment for NPVH typically depends on subjective scales and instrumental assessments of voice (Patel et al., 2018). Subjective scales include the clinician-administered Consensus Auditory-Perceptual Evaluation of Voice (CAPE-V; Kempster et al., 2009) and patient-reported outcomes, such as the Voice-Related Quality of Life questionnaire (V-RQOL; Hogikyan & Sethuraman, 1999) and Voice Handicap Index (VHI; Jacobson et al., 1997). Subjective instrumental assessments include visual perception–based analysis of laryngeal videostroboscopy. As a complement to these subjective voice assessments, objective measurements can enable quantification of voice improvement after therapy (Toles et al., 2022).

In 2012, the American Speech-Language-Hearing Association (ASHA) tasked an Expert Panel to Develop a Recommended Protocol for Instrumental Assessment of Vocal Function (Patel et al., 2018). The panel focused on laryngeal endoscopic imaging, acoustic assessment, and aerodynamic measurement as three crucial instrumental methods for clinical voice assessment. A standard, minimal set of acoustic and aerodynamic measures was recommended that could also provide the basic parameters necessary to derive additional voice metrics such as vocal efficiency (VE). VE methods combine acoustic and aerodynamic measures to estimate the efficiency, or economy, of energy transduction of aerodynamic power (AP) to acoustic power during voice production (Schutte, 1992; Titze, 1992). As an energy transducer, the larynx converts aerodynamic energy generated by the lungs into acoustic energy perceived by listeners, that is, an estimate of respiratory-laryngeal coordination. AP is assessed through estimating subglottal pressure (Ps) and glottal airflow (AFLOW). Acoustic power is measured from the oral sound pressure level (SPL). VE metrics are of interest, in part, because studies have shown that the individual measures underlying VE can be nonspecific to NPVH. For example, 52% of females with NPVH in one study presented with typical Ps (Huth et al., 2020), and 55% of individuals with NPVH in another study presented with typical AFLOW (Belsky et al., 2021). And even SPL can display intraindividual differences across repeated recording sessions (Holmberg et al., 1994). Thus, Ps, SPL, or AFLOW alone might not accurately capture vocal function related to NPVH behavior. However, combining these objective metrics into ratios between acoustic output and aerodynamic input, that is, VE, may improve upon the quantitative assessment of NPVH. In fact, VE has already been shown to be sensitive to treatment-related effects in different types of voice disorders (Bullock et al., 2023; Toles et al., 2022).

Traditional VE is a ratio between vocal intensity and AP, where vocal intensity is proportional to the square of the sound pressure level (Schutte, 1992; Titze, 1992). Although the traditional measure of VE is promising for capturing voice changes over time, it still has some limitations. Some scientists have simplified the traditional VE ratio to the ratio of acoustic SPL to AP (SPL/AP; Bullock et al., 2023; Grillo & Verdolini, 2008; Toles et al., 2022). Two additional simplified VE metrics are the ratio of average SPL to average Ps (SPL/Ps) and the ratio of average SPL to average airflow (SPL/AFLOW; Bullock et al., 2023; Espinoza et al., 2017; Toles et al., 2022). Previous studies have shown that average airflow measures can demonstrate more variability using current measurement methods compared to SPL and Ps during the standard p-vowel syllable string required to indirectly estimate Ps in the clinic (Toles et al., 2022). Therefore, the simplified ratio of SPL/Ps, where the airflow parameter is removed, has been proposed. Compared to other VE measurements that involve glottal airflow, the SPL/Ps ratio has been found to significantly improve differentiation between individuals with vocal hyperfunction from individuals with healthy voice and is less influenced by vocal loudness (Espinoza et al., 2017; Grillo & Verdolini, 2008; Toles et al., 2022). Several studies have found that after surgical removal of phonotraumatic lesions (vocal fold nodules or polyps), individuals' VE increased significantly (Toles et al., 2022; Zeitels et al., 2002). Toles et al. (2022) also found that, among all the different methods for measuring VE, SPL/Ps showed the least within-subject variability. A significantly lower SPL/Ps ratio is also exhibited in individuals with glottal cancer compared to vocally healthy individuals (Zeitels et al., 2002). Therefore, the SPL/Ps ratio has been suggested to be ideal for assessing hyperfunctional voice disorders, including changes that occur as a result of treatment (Mehta & Hillman, 2007).

Although VE metrics have demonstrated promise for assessing vocal function and detecting treatment effects in several types of voice disorders, the sensitivity of VE in individuals with NPVH is yet to be determined. In this study, we aimed to quantitatively describe how accurately VE ratios reflect therapy-related changes in the NPVH population.

## Method

### Participants

In this study, there were two sources of data. The first source of data was the clinical database of the Massachusetts General Hospital Center for Laryngeal Surgery and Voice Rehabilitation (MGH Voice Center). The second source of data was from individuals who participated in research studies at the Vocal Hyperfunction Clinical Research Center (research database), also at the MGH Voice Center. All research participants provided written informed consent for the study. The protocols supporting this study were approved by the institutional review board at Mass General Brigham (Protocol Numbers 2008P000616 and 2011P002376). Data were obtained for individuals with NPVH who received an official diagnosis of primary muscle tension dysphonia and who completed pre- and posttreatment vocal function evaluations at the MGH Voice Center. Vocal function was evaluated based on (a) case history, (b) patient completion of the V-RQOL, (c) auditory-perceptual evaluation using the CAPE-V from the treating clinician, (d) laryngeal videoendoscopy with stroboscopy, (e) acoustic assessment, and (f) aerodynamic assessment (Patel et al., 2018). Individuals diagnosed with NPVH were included in the study, if they also had secondary diagnoses of laryngopharyngeal reflux and gastroesophageal reflux disease. However, individuals with NPVH who had other complications of structural and/or neurological disorders were excluded from the study, including spasmodic dysphonia/laryngeal dystonia, presbylarynx, torticollis of the sternocleidomastoid muscle, laryngitis, benign vocal fold lesions, dysphagia, and a presentation following thyroidectomy procedures (Kridgen et al., 2021).

**Figure 1.**
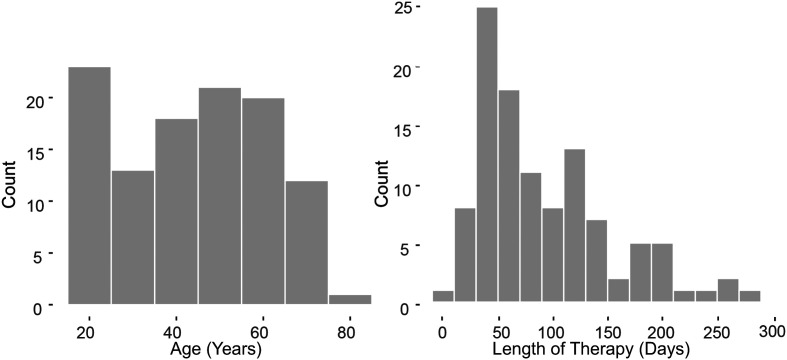
Demographic summary of the 108 individuals in the study who were diagnosed with primary muscle tension dysphonia that is associated with nonphonotraumatic vocal hyperfunction behavior.

As a result of the inclusion and exclusion criteria, 80 females and 28 males with NPVH were included in the study. Among these participants, 65 females and 23 males were from the clinical database, and 15 females and five males were from the research database. The age range of the patient cohort was 16 to 77 years old (*M*_age_ = 43.6, *SD*_age_ = 17.6, *Mdn*_age_ = 43.6, *IQR*_age_ = 31.3). The therapy duration ranged from 2 days to 277 days (*M*_duration_ = 92.9, *SD*_duration_ = 60.0, *Mdn*_duration_ = 73.0, *IQR*_duration_ = 76.3; mostly within 4–6 weeks, with outliers seen in Figure 1). The demographic summary of the patient participants is shown in Figure 1. Additionally, 181 females and 27 males from the research database who were vocally healthy served as controls to better interpret the VE measures in the patient cohort. The normal vocal status of all participants in the control group was verified via interview and a laryngeal stroboscopic examination by a voice-specialized speech-language pathologist. The age of the control cohort ranged from 18 to 65 years old (*M*_age_ = 29.4, *SD*_age_ = 12.3).

### Clinical Outcome Scales

Voice therapy was considered successful if each patient and their treating speech-language pathologist jointly agreed that therapy goals were met. As a secondary analysis, when available, CAPE-V and V-RQOL scales were used to document subjective changes before and after voice therapy. The CAPE-V was performed by a single treating clinician following standard procedures to assess voice quality attributes, including overall severity, roughness, breathiness, strain, pitch, and loudness based on the clinician's auditory perception (Kempster et al., 2009). CAPE-V scores ranged from 0 to 100, with higher scores indicating a more severe disruption in a given attribute. For the V-RQOL, patients were asked to self-rate their perception of their voice and its impact on their daily life using ten questions, leading to an overall measure ranging from 0 to 100, with higher scores indicating better vocal functioning during daily life (Hogikyan & Sethuraman, 1999). The impact of their voice disorder on social–emotional and physical functioning attributes makes up two subscales of the V-RQOL.

There were missing data points since many of these measures were obtained during the course of standard clinical care, during which all measures were not necessarily obtained before and after voice therapy. Group-wide CAPE-V and V-RQOL scores pre- and postvoice therapy for the patient group are presented in Table 1. Almost all patient participants (*n* = 105) had CAPE-V scores available before therapy, and more than half of the participants had CAPE-V scores available after therapy (*n* = 63). Almost all patient participants (*n* = 107) completed the V-RQOL before therapy, and more than half of the participants completed V-RQOL after therapy (*n* = 72). These subjective scales are reported only for the purpose of generally describing the severity level of the patient group, not for statistical analysis or results reporting.

**Table 1.** Documentation of the Voice-Related Quality of Life questionnaire (V-RQOL) and Consensus Auditory-Perceptual Evaluation of Voice (CAPE-V) evaluations pre– and post–voice therapy.

Perceptual assessment scale	Pretherapy *M*(*SD*)	Posttherapy *M*(*SD*)
V-RQOL		
Social–emotional	69.34 (26.35)	92.70 (11.21)
Physical functioning	64.99 (21.35)	88.86 (11.20)
Total score	66.88 (21.11)	90.41 (10.17)
CAPE-V		
Overall severity	30.60 (24.80)	8.41 (7.42)
Roughness	18.40 (17.57)	6.48 (6.91)
Breathiness	14.26 (20.06)	2.09 (4.20)
Strain	21.84 (22.03)	5.31 (5.97)
Pitch	11.52 (20.30)	1.74 (6.61)
Loudness	13.09 (22.39)	1.71 (5.40)

*Note.* Availability of posttherapy ratings: V-RQOL (*n* = 72), CAPE-V (*n* = 63).

### Objective Acoustic and Aerodynamic Voice Evaluation

For participants in the clinical database, acoustic and aerodynamic data were obtained with the Phonatory Aerodynamic System (KayPENTAX). The system consisted of a head-mounted omnidirectional microphone placed at a distance of 15 cm from the mouth for acoustic measurement of SPL, a face mask with a built-in low-bandwidth airflow sensor for average oral airflow measurement, and an intraoral tube attached to a pressure transducer to estimate average subglottal air pressure. For participants who participated in research studies at the Vocal Hyperfunction Clinical Research Center, a high-bandwidth airflow sensor was used with a circumferentially vented pneumotachograph face mask (PT-2E, Glottal Enterprises). A low-bandwidth air pressure sensor (PT-25, Glottal Enterprises) was connected to a similar intraoral tube for Ps estimation. All participants were instructed to produce the consonant–vowel utterance /pa:pa:pa:pa:pa:/ at both comfortable and loud conditions following the standard protocol (Patel et al., 2018; Rothenberg, 1973). The consonant–vowel utterance was repeated three times for each loudness condition, before and after treatment. The /pa/ syllables (ignoring the first and last transient syllables) were used to calculate the mean Ps, mean airflow (AFLOW), and mean SPL for each treatment stage and loudness condition. The four versions of VE ratios were calculated from these three values.

### VE Calculation

Traditional VE is a unitless value presented in terms of parts per million (ppm). It is calculated with the following formula, which is provided by the PENTAX Phonatory Aerodynamic System software manual (Bullock et al., 2023; Toles et al., 2022):

$$\text{Traditional Vocal Efficiency}\;\left(\mathrm{ppm}\right)=\frac{1.4137\times 10^{-7}\times 10^{\frac{\mathrm{SPL}}{10}}}{0.09806\times \mathrm{Ps}\times \text{AFLOW}}$$ (1)

The ratio of acoustic to AP is defined as follows in units of (dB SPL)/(cm H_2_O * L/s; Bullock et al., 2023; Grillo & Verdolini, 2008; Toles et al., 2022):

$$\mathrm{SPL}/\mathrm{AP}\left(\frac{\mathrm{dB}\;\mathrm{SPL}}{\left[\mathrm{cm}H_{2}O\right]\left[L/s\right]}\right)=\frac{\mathrm{SPL}}{\mathrm{Ps}\times \text{AFLOW}}$$ (2)

The ratio of SPL to Ps is defined as follows, in units of (dB SPL)/(cm H_2_O; Bullock et al., 2023; Toles et al., 2022):

$$\mathrm{SPL}/\mathrm{Ps}\left(\frac{\mathrm{dB}\;\mathrm{SPL}}{\mathrm{cm}H_{2}O}\right)=\frac{\mathrm{SPL}}{\mathrm{Ps}}$$ (3)

Finally, the ratio of SPL to AFLOW was defined as follows, in units of (dB SPL)/(L/s; Bullock et al., 2023; Toles et al., 2022):

$$\mathrm{SPL}/\text{AFLOW}\left(\frac{\mathrm{dB}\;\mathrm{SPL}}{L/s}\right)=\frac{\mathrm{SPL}}{\text{AFLOW}}$$ (4)

### Statistical Analysis

All statistical analyses were performed using R (Version 4.4.1). Individuals with VE values that were 3 *SD*s below or above the mean within a given sex and loudness condition were considered outliers and were removed from statistical analyses of that VE calculation. Table 2 summarizes the number of patients included for each VE calculation following outlier removal within each sex and loudness condition. Mixed-design analysis of variance (ANOVA) models were conducted to investigate whether sex, treatment stage (pretherapy, posttherapy, and control), and loudness condition (soft, comfortable, loud) had a statistically significant main and/or interaction effect for each of the four VE measures. The vocally healthy control group acted as an external reference to determine whether posttherapy changes in VE migrated toward normative ranges.

**Table 2.** Number of participants after outlier removal for each vocal efficiency (VE) calculation, loudness condition, and sex.

Loudness	Sex	Traditional VE	SPL/AP	SPL/Ps	SPL/AFLOW
Comfortable	Female	72	72	76	72
	Male	27	27	27	25
Loud	Female	72	73	76	72
	Male	27	25	27	25

*Note.* There were 80 females and 28 males before outlier removal. SPL/AP = sound pressure level over aerodynamic power; SPL/Ps = sound pressure level over subglottal pressure; SPL/AFLOW = sound pressure level over airflow.

The data were analyzed using a mixed-design ANOVA with within-subject factors of loudness and treatment stage and between-subject factor of sex. Prior to analysis, residuals for normality were tested using the Shapiro–Wilk test, and homogeneity of variances was assessed with Levene's test (*p* < .001). The residuals exhibited violations of normality for all four VEs and homogeneity of variances for traditional VE and SPL/Ps. To address these issues, we applied robust heteroskedasticity-consistent standard errors (HC-3) that adjust for both nonnormality and unequal variances, ensuring more reliable statistical results despite these violations. For the omnibus test, *p* values less than .05 were considered statistically significant.

Post hoc paired and independent sample comparisons were conducted to quantify the direction of significant differences across groups and treatment stages. Assumptions for parametric testing—normality and homogeneity of variance—were assessed using the Shapiro–Wilk test and *F* test, respectively. As several comparisons violated these assumptions, and to ensure consistency across all statistical analyses, non-parametric tests were applied for all group comparisons, regardless of whether parametric assumptions were met. Specifically, comparisons between pre- and posttherapy were treated as repeated measurements and analyzed using the Wilcoxon signed-rank test. Comparisons between pretherapy and control groups, as well as posttherapy and control groups, involved independent samples and were analyzed using the Wilcoxon rank-sum test (i.e., Mann–Whitney *U* test). In addition to reporting test statistics and *p* values, we also calculated effect sizes using the *r* statistic to quantify the magnitude of observed effects. Following conventional benchmarks, *r* values of approximately .1, .3, and .5 were interpreted as small, medium, and large effects, respectively (Cohen, 1988). A conservative Bonferroni correction was applied to the threshold of statistical significance (α = .05/3 ≈ .01667) to account for the following three multiple comparisons: pretherapy versus control, posttherapy versus control, and pretherapy versus posttherapy.

For the statistical tests comparing patient and control groups, positive (negative) values of *r* indicated larger (smaller) values for a given measure in the control group. For the treatment-related tests in the patient group, positive (negative) values of *r* indicated larger (smaller) values following voice therapy. Boxplots were generated to visualize treatment-related trends and any migration toward normative values, where the horizontal line inside the box represented the median, the lower and upper borders represented the first and third quartiles, lines extended to 1.5 *SD*s away from the first and third quartiles, and dots represented the data points between 1.5 and 3 *SD*s away from the first and third quartiles.

## Result

### Groupwide Statistical Analysis of VE

Across the whole group, linear models were fit for each of the four VE calculations with robust standard errors (HC-3) to account for heteroskedasticity to examine the effects of sex, treatment stage, and loudness condition on VE. No significant main effects of treatment were observed for any VE metric (traditional VE: *p* = .65; SPL/AP: *p* = .70; SPL/Ps: *p* = .30; SPL/AFLOW: *p* = .76). Two-way interactions involving treatment (i.e., treatment × sex; treatment × loudness) were not significant for all VE measures. A significant three-way interaction (sex × treatment × loudness) was identified for SPL/AFLOW (*p* = .02), whereas the same interaction was nonsignificant for the remaining measures.

Table 3 reports the results of the mixed-design ANOVA models that further corroborated the absence of significant treatment effects. For SPL/Ps, a marginal interaction between treatment stage and loudness condition was detected (*p* = .046); however, follow-up pairwise comparisons revealed no significant differences between pre- and posttreatment at either loudness condition (comfortable: *p* = .20; loud: *p* = .60). For SPL/AFLOW, although the three-way interaction reached statistical significance (*p* = .031), post hoc comparisons indicated no statistically significant changes from pre- to posttreatment within any sex and loudness condition (*p* > .08).

**Table 3.** Results for each mixed-design analysis of variance modeling the relationship between each vocal efficiency (VE) measure and sex, treatment stage (pretherapy, posttherapy, control), loudness condition (soft, comfortable, loud), and the interaction among them.

VE measure	Effect	*F*(1, 97)	*p*	ƞ^2^
Traditional VE				
	Sex	2.47	.12	.009
	Treatment	3.78	.055	.008
	Loudness	30.80	< .001**	.078
	Sex * Treatment	0.002	.97	< .001
	Sex * Loudness	4.11	.045*	.011
	Treatment * Loudness	1.46	.23	.002
	Sex * Treatment * Loudness	0.81	.37	.001
SPL/AP		*F*(1, 96)	*p*	ƞ^2^
	Sex	0.18	.67	< .001
	Treatment	1.46	.23	.005
	Loudness	15.06	< .001**	.019
	Sex * Treatment	3.04	.084	.01
	Sex * Loudness	3.02	.085	.004
	Treatment * Loudness	1.41	.24	.001
	Sex * Treatment * Loudness	0.13	.72	< .001
SPL/Ps		*F*(1, 101)	*p*	ƞ^2^
	Sex	6.25	.014*	.032
	Treatment	0.01	.93	< .001
	Loudness	75.00	< .001**	.16
	Sex * Treatment	0.60	.44	< .001
	Sex * Loudness	1.40	.24	.004
	Treatment * Loudness	4.09	.046*	.002
	Sex * Treatment * Loudness	0.40	.53	< .001
SPL/AFLOW		*F*(1, 95)	*p*	ƞ^2^
	Sex	14.43	< .001**	.055
	Treatment	0.20	.65	< .001
	Loudness	0.22	.64	< .001
	Sex * Treatment	0.39	.53	.001
	Sex * Loudness	3.07	.083	.007
	Treatment * Loudness	0.11	.75	< .001
	Sex * Treatment * Loudness	4.81	.031*	.008

*Note.* *F* value (*F*), *p* value (*p*), and eta-squared values (ƞ^2^) are reported for each measure. SPL/AP = sound pressure level over aerodynamic power; SPL/Ps = sound pressure level over subglottal pressure; SPL/AFLOW = sound pressure level over airflow.

* *p* < .05.

** *p* < .01.

### Exploratory Subgroup Analysis of VE

Based on the results of these omnibus tests, subsequent exploratory statistical analyses were performed on subgroups of the study participants with NPVH. First, separate models were run for comfortable and loud conditions (due to the strong effect of loudness condition in Table 3). Second, due to the known heterogeneous presentation of vocal function associated with NPVH, it was hypothesized that NPVH subgroups would show VE-related treatment effects that were not shown when analyzing the entire NPVH group together. To evaluate this hypothesis, patients were evenly divided into three subgroups based on their pretherapy VE values. The three NPVH subgroups were created according to values for each VE metric to yield low (lowest third), middle (middle third), and high (highest third) subgroups within each loudness condition for males and females separately. For this investigation on subgroup-based treatment effects, outlier values were removed prior to statistical analyses to allow for comparisons of VE. Of interest is whether VE would demonstrate treatment effects within NPVH subgroups since no groupwide treatment effects were found in Table 3.

**Figure 2.**
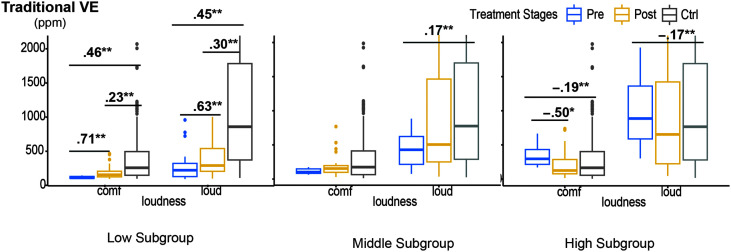
Values for traditional vocal efficiency (VE) in females in low, middle, and high subgroups before and after therapy. Blue boxes are patients before therapy, yellow boxes are patients after therapy, and gray boxes are the vocally healthy control group. The numbers on bars are *r* value effect sizes for groups with significant differences. *p < .05/3, **p < .01/3. Unit: ppm = parts per million; comf = comfortable; Pre = pretherapy; Post = posttherapy; Ctrl = control group.

**Figure 3.**
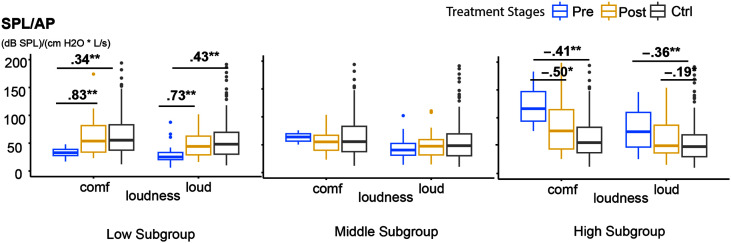
Values for sound pressure level over aerodynamic power (SPL/AP) in females in low, middle, and high subgroups before and after therapy. Blue boxes are patients before therapy, yellow boxes are patients after therapy, and gray boxes are the vocally healthy control group. The numbers on bars are *r* value effect sizes for groups with significant differences. *p < .05/3, **p < .01/3. Unit: dB SPL/(cm H_2_O * L/s). comf = comfortable; Pre = pretherapy; Post = posttherapy; Ctrl = control group; Ctrl = control group.

**Figure 4.**
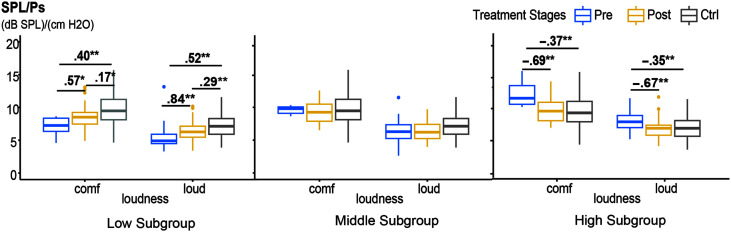
Values for sound pressure level over subglottal pressure (SPL/Ps) in females in low, middle, and high subgroups before and after therapy. Blue boxes are patients before therapy, yellow boxes are patients after therapy, and gray boxes are the vocally healthy control group. The numbers on bars are *r* value effect sizes for groups with significant differences. *p < .05/3, **p < .01/3. Unit: (dB SPL)/(cm H_2_O). comf = comfortable; Pre = pretherapy; Post = posttherapy; Ctrl = control group; Ctrl = control group.

**Figure 5.**
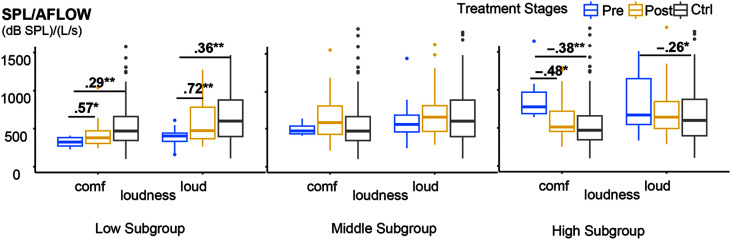
Values for sound pressure level over airflow (SPL/AFLOW) in females in low, middle, and high subgroups before and after therapy. Blue boxes are patients before therapy, yellow boxes are patients after therapy, and gray boxes are patients in the vocally healthy control group. The numbers on bars are *r* value effect sizes for groups with significant differences. *p < .05/3, **p < .01/3. Unit: (dB SPL)/(L/s). comf = comfortable; Pre = pretherapy; Post = posttherapy; Ctrl = control group; Ctrl = control group.

Tables 4–7 tabulate the detailed descriptive statistics and all the NPVH subgroup values before and after therapy in the female and male cohorts for each of the four VE metrics, respectively. Due to the small sample size and generally nonsignificant results in the male cohort, Figures 2–5 show box-whisker plots for each of the four VE metrics, respectively, within each of the three female NPVH subgroups before and after therapy compared with the vocally healthy control group. The results of post hoc comparisons—Wilcoxon signed-rank tests for pre- versus posttherapy (paired samples) and Wilcoxon rank-sum tests for therapy versus control groups (independent samples)—are displayed in the tables and figures. These nonparametric tests were used due to violations of normality and homogeneity of variance assumptions.

**Table 4.** Nonphonotraumatic vocal hyperfunction patient subgroup changes in traditional vocal efficiency before to after therapy compared with the vocally healthy control group (plotted in Figure 2).

Subgroup	*N*	Pre-*M* (*SD*)	Post-*M* (*SD*)	Pre–Post *p*	Pre–Post *r*	Ctrl *N*	Ctrl *M* (*SD*)	Pre-Ctrl *p*	Pre-Ctrl *r*	Post-Ctrl *p*	Post-Ctrl *r*
Female, comf											
High	24	340.43 (154.71)	220.20 (214.58)	**.014** *	−.50	179	315.51 (394.06)	**.0058** *	−.19	.51	.05
Middle	24	103.67 (38.46)	186.26 (174.77)	.023	.46			.034	.15	.46	.05
Low	24	22.55 (16.61)	100.68 (107.30)	**< .001** **	.71			**< .001** **	.46	**.0013** **	.23
Female, loud											
High	24	1,388.93 (893.45)	1,477.10 (1,529.44)	.64	.01	178	1,284.11 (1,710.71)	**.014** *	−.17	.092	−.12
Middle	24	425.61 (125.14)	799.52 (1,365.90)	.92	.02			**< .016** *	.17	.020	.16
Low	24	94.44 (71.00)	415.09 (860.52)	**.0012** **	.63			**< .001** **	.45	**< .001** **	.30
Male, comf											
High	9	237.13 (85.11)	175.64 (78.51)	.098	−.57	26	270.87 (259.01)	.70	.07	.47	.13
Middle	9	85.70 (23.34)	197.29 (228.21)	.25	.42			.016*	.40	.32	.17
Low	9	32.45 (16.60)	338.23 (314.54)	.019	.77			**< .001** **	.60	.54	−.11
Male, loud											
High	9	746.19 (368.90)	502.62 (335.71)	.098	−.57	25	597 (579.64)	.15	−.25	.88	.03
Middle	9	316.95 (68.75)	743.24 (942.58)	.73	.14			.32	.18	.76	− .06
Low	9	87.61 (38.03)	374.43 (258.12)	.039	.69			**< .001** **	.69	.42	.14

*Note.* Unit: parts per million. Bold text indicates statistically significant *p* values. *N* = number of individuals in the group; Pre = pretherapy; Post = posttherapy; Ctrl = control group; comf = comfortable.

* *p* < .05/3.

** *p* < .01/3.

**Table 5.** Nonphonotraumatic vocal hyperfunction patient subgroup changes in sound pressure level over aerodynamic power before to after therapy compared with the vocally healthy control group (plotted in Figure 3).

Subgroup	*N*	Pre-*M* (*SD*)	Post-*M* (*SD*)	Pre–Post *p*	Pre–Post *r*	Ctrl *N*	Ctrl *M* (*SD*)	Pre-Ctrl *p*	Pre-Ctrl *r*	Post-Ctrl *p*	Post-Ctrl *r*
Female, comf											
High	24	130.50 (59.23)	87.08 (49.06)	**.014** *	−.50	176	69.05 (52.67)	**< .001** **	−.41	.027	−.16
Middle	24	63.57 (7.98)	62.83 (44.37)	.20	−.26			.081	.12	.81	.02
Low	24	33.18 (8.84)	65.97 (47.44)	**< .001** **	.83			**< .001** **	.34	.73	.03
Female, loud											
High	24	98.05 (37.31)	109.48 (123.22)	.26	.23	177	64.90 (61.90)	**< .001** **	−.36	**.0067** *	−.19
Middle	25	41.14 (7.38)	48.03 (19.14)	.11	.33			.14	.10	.66	.03
Low	25	21.48 (6.64)	58.52 (44.11)	**< .001** **	.73			**< .001** **	.43	.019	.17
Male, comf											
High	9	225.80 (342.98)	106.98 (55.57)	.74	−.15	26	50.69 (36.84)	**< .001** **	−.54	**< .001** **	−.54
Middle	9	47.76 (7.72)	49.24 (26.46)	.55	.25			.35	.17	.71	.07
Low	9	24.17 (8.20)	34.06 (19.79)	.020	.77			**.0031** **	.49	.073	.31
Male, loud											
High	8	122.00 (153.17)	33.14 (15.51)	**.0078** *	−.89	25	30.08 (16.48)	**< .001** **	−.68	.52	−.12
Middle	8	38.15 (5.76)	45.69 (14.22)	.20	.50			.036	−.37	**.015** *	−.42
Low	9	13.96 (5.75)	17.16 (10.71)	.50	.26			**.0026** **	.50	**.012** *	.43

*Note.* Unit: dB SPL/(cm H_2_O * L/s). Bold text indicates statistically significant *p* values. *N* = number of individuals in the group; Pre = pretherapy; Post = posttherapy; Ctrl = control group; comf = comfortable.

* *p* < .05/3.

** *p* < .01/3.

**Table 6.** Nonphonotraumatic vocal hyperfunction patient subgroup changes in SPL/Ps before to after therapy compared with the vocally healthy control group (plotted in Figure 4).

Subgroup	*N*	Pre-*M* (*SD*)	Post-*M* (*SD*)	Pre–Post *p*	Pre–Post *r*	Ctrl *N*	Ctrl *M* (*SD*)	Pre-Ctrl *p*	Pre-Ctrl *r*	Post-Ctrl *p*	Post-Ctrl *r*
Female, comf											
High	26	12.13 (1.65)	9.96 (1.97)	**< .001** **	−.69	177	9.71 (2.03)	**< .001** **	−.37	.59	−.04
Middle	25	9.63 (0.52)	9.31 (1.80)	.38	−.18			.65	.03	.43	.06
Low	25	7.19 (1.19)	8.63 (2.10)	**.0034** *	.57			**< .001** **	.40	**.016** *	.17
Female, loud											
High	26	9.01 (1.46)	7.40 (1.90)	**< .001** **	−.67	178	7.20 (1.61)	**< .001** **	−.35	.56	−.04
Middle	25	6.45 (0.68)	6.80 (1.45)	.40	.18			< .022	.16	.16	.10
Low	25	4.47 (0.75)	5.79 (1.41)	**< .001** **	.84			**< .001** **	.52	**< .001** **	.29
Male, comf											
High	9	13.66 (2.09)	12.08 (1.78)	.13	−.53	25	9.44 (1.64)	**< .001** **	−.70	**< .001** **	−.55
Middle	9	9.86 (0.48)	9.66 (1.62)	.91	−.06			.32	−.18	.67	−.08
Low	9	7.33 (1.29)	8.59 (2.19)	.027	.73			**< .001** **	.55	.44	.14
Male, loud											
High	9	12.07 (3.76)	11.99 (5.56)	.57	−.22	25	6.63 (1.43)	**< .001** **	−.73	**< .001** **	−.56
Middle	9	7.03 (0.91)	7.77 (3.15)	.91	.06			.38	−.16	.49	−.12
Low	9	4.31 (0.59)	5.10 (0.98)	.074	.61			**< .001** **	.64	**.0055** *	.47

*Note.* Unit: (dB SPL)/(cm H_2_O). Bold text indicates statistically significant *p* values. *N* = number of individuals in the group; Pre = pretherapy; Post = posttherapy; Ctrl = control group; comf = comfortable.

* *p* < .05/3.

** *p* < .01/3.

**Table 7.** Nonphonotraumatic vocal hyperfunction patient subgroup changes in sound pressure level over airflow before to after therapy compared with the vocally healthy control group (plotted in Figure 5).

Subgroup	*N*	Pre *M* (*SD*)	Post *M* (*SD*)	Pre–Post *p*	Pre–Post *r*	Ctrl *N*	Ctrl *M* (*SD*)	Pre-Ctrl *p*	Pre-Ctrl *r*	Post-Ctrl *p*	Post-Ctrl *r*
Female, comf											
High	24	990.11 (517.42)	632.12 (290.62)	**.016** *	−.48	178	585.94 (449.89)	**< .001** **	−.38	.076	−.13
Middle	24	468.54 (65.46)	634.57 (323.62)	.053	.40			.61	.04	.11	−.11
Low	24	326.82 (62.56)	471.67 (337.01)	**.0044** *	.57			**< .001** **	.29	.075	.13
Female, loud											
High	24	1037.29 (494.63)	969.04 (901.43)	.025	−.46	179	894.85 (1,170.12)	**< .001** **	−.26	.078	−.12
Middle	24	507.85 (53.15)	754.08 (572.05)	.019	.47			.10	.12	.59	.04
Low	24	334.66 (83.39)	539.06 (251.31)	**< .001** **	.72			**< .001** **	.36	.072	.17
Male, comf											
High	8	596.97 (196.78)	748.99 (434.19)	.64	.20	26	411.78 (214.24)	**.0060** *	−.46	**.0069** *	−.45
Middle	8	367.08 (18.08)	377.37 (130.98)	.84	.10			.59	.10	.86	.04
Low	9	241.44 (71.84)	344.11 (193.69)	.039	.69			**.0027** **	.49	.20	.22
Male, loud											
High	8	750.80 (260.24)	430.51 (177.50)	.039	−.74	25	394.14 (164.07)	**< .001** **	−.59	.50	−.12
Middle	8	375.44 (54.59)	357.24 (66.51)	.74	−.15			.79	.05	.98	.01
Low	9	212.06 (53.25)	217.15 (70.59)	.73	.14			**< .001** **	.59	**< .001** **	.56

*Note.* Unit: (dB SPL)/(L/s). Bold text indicates statistically significant *p* values. *N* = number of individuals in the group; Pre = pretherapy; Post = posttherapy; Ctrl = control group; comf = comfortable.

* *p* < .05/3.

** *p* < .01/3.

### Traditional VE

Table 4 and Figure 2 report significant treatment effects in the low traditional VE female subgroup in both comfortable and loud conditions and in the high traditional VE subgroup in the comfortable condition. No significant therapy-related changes in traditional VE were observed for the middle VE subgroups in both loudness conditions and for the high VE subgroup in the loud condition.

*Comfortable condition*. A pre- to posttherapy increase was noted in the low VE subgroup, with a large effect size (*r* = .71, *p* < .001). Although posttherapy VE values were still significantly different from the control group (*r* = .23, *p* = .0013), the posttherapy values migrated toward those in the normative range (*M*_pretherapy_ = 22.55, *M*_posttherapy_ = 100.68, *M*_control_ = 315.51). In the high VE subgroup, a significant pre- to postdecrease was noted with a large effect size (*r* = −.50, *p* = .014). The difference between posttherapy VE and the control group at comfortable loudness in the high subgroup was not significant (*M*_pretherapy_ = 340.43, *M*_posttherapy_ = 220.20, *M*_control_ = 315.51).

*Loud condition*. In the low VE subgroup, a significant pre- to postincrease was noted with a large effect size (*r* = .63, *p* = .0012). Even though the difference between posttherapy VE and the control group this subgroup was still significant (*r* = .30, *p* < .001), the posttherapy value migrated toward the normative range (*M*_pretherapy_ = 94.44, *M*_posttherapy_ = 415.09, *M*_control_ = 1,284.11).

### SPL/AP

Table 5 and Figure 3 report significant treatment effects for low and high SPL/AP female subgroups in the comfortable condition and for the low SPL/AP subgroup in the loud condition. No significant therapy-related changes in SPL/AP were observed for the middle subgroups.

*Comfortable condition*. A significant pre- to posttherapy increase was observed in the low SPL/AP subgroup, with a large effect size (*r* = .83, *p* < .001). Importantly, posttherapy SPL/AP values were not significantly different from the control group, demonstrating a migration toward and complete recovery to values in the normative range (*M*_pretherapy_ = 33.18, *M*_posttherapy_ = 65.97, *M*_control_ = 69.05). A significant pre- to posttherapy decrease was noted in the high SPL/AP subgroup, with a large effect size (*r* = −.50, *p* = .014). Thus, in the case of the high subgroup, SPL/AP values decreased posttherapy in the direction of and complete recovery to values in the normative range (*M*_pretherapy_ = 130.50, *M*_posttherapy_ = 87.08, *M*_control_ = 69.05).

*Loud condition*. In the low SPL/AP subgroup, a significant pre- to posttherapy increase was noted with a large effect size (*r* = .73, *p* < .001) and complete recovery to normative values posttherapy (*M*_pretherapy_ = 21.48, *M*_posttherapy_ = 58.52, *M*_control_ = 64.90). A posttherapy decrease in the high SPL/AP subgroup was not observed.

### SPL/Ps

Table 6 and Figure 4 report significant treatment effects for low and high SPL/Ps female subgroups in both loudness conditions. No significant therapy-related changes in SPL/Ps were observed for the middle pretherapy subgroups.

*Comfortable condition*. A significant pre- to posttherapy increase was observed in the low SPL/Ps subgroup, with a large effect size (*r* = .57, *p* = .0034). Posttherapy SPL/Ps values were significantly different from the control group, demonstrating a migration toward and partial recovery to values in the normative range (*M*_pretherapy_ = 7.19, *M*_posttherapy_ = 8.63, *M*_control_ = 9.71). A large pre- to posttherapy decrease was noted in the high SPL/Ps subgroup (*r* = −.69, *p* < .001); posttherapy SPL/Ps values not significantly different from the control group. Thus, SPL/Ps values in the high subgroup decreased posttherapy in the direction of and with complete recovery to values in the normative range (*M*_pretherapy_ = 12.13, *M*_posttherapy_ = 9.96, *M*_control_ = 9.71).

*Loud condition*. In the low SPL/Ps subgroup, a significant pre- to posttherapy increase was noted with a large effect size (*r* = .84, *p* < .001) and migration toward normative values (*M*_pretherapy_ = 4.47, *M*_posttherapy_ = 5.79, *M*_control_ = 7.20). In contrast with traditional VE and SPL/AP, a large pre- to posttherapy decrease was noted in the high SPL/Ps subgroup in the loud condition (*r* = −.67, *p* < .001). SPL/Ps values posttherapy were statistically similar to those in the control group (*M*_pretherapy_ = 9.01, *M*_posttherapy_ = 7.40, *M*_control_ = 7.20).

### SPL/AFLOW

Table 7 and Figure 5 report significant treatment effects for low and high SPL/AFLOW female subgroups in the comfortable condition and for the low SPL/AFLOW subgroup in the loud condition. No significant therapy-related changes in SPL/AFLOW were observed for the middle pretherapy subgroups.

*Comfortable condition*. A significant pre- to posttherapy increase was noted in the low SPL/AFLOW subgroup, with a large effect size (*r* = .57, *p* = .0044) and complete recovery to values in the normative range (comfortable: *M*_pretherapy_ = 326.82, *M*_posttherapy_ = 471.67, *M*_control_ = 585.94). A significant pre- to posttherapy decrease was noted in the high SPL/AFLOW subgroup, with a large effect size (*r* = −.48, *p* = .016). Thus, in the case of the high subgroup, SPL/AFLOW values decreased posttherapy in the direction of and complete recovery to values in the normative range (*M*_pretherapy_ = 990.11, *M*_posttherapy_ = 632.12, *M*_control_ = 585.94).

*Loud condition*. A significant pre- to posttherapy increase was noted in the low SPL/AFLOW subgroup, with a large effect size (*r* = .72, *p* < .001) and complete recovery to values in the normative range (*M*_pretherapy_ = 334.66, *M*_posttherapy_ = 539.06, *M*_control_ = 894.85). A posttherapy decrease in the high SPL/AFLOW subgroup was not observed.

## Discussion

This study investigated the ability of four methods for calculating VE to capture the effects of voice therapy in individuals with NPVH, with the aim of finding VE measures that could complement traditional subjective voice assessments. Initial statistical testing using an omnibus ANOVA revealed negligible treatment-related effects for the four studied VE metrics. Subgrouping the patients according to pretherapy values for certain VE measures revealed statistically significant effects of treatment (pre- to posttherapy) and, further, that the direction of these effects was toward VE values exhibited by the vocally healthy control group. Potential limitations due to the statistical approach are provided below in the Limitations section.

Tables 4–7 reported the pre- to posttherapy changes for each VE metric within each NPVH subgroup (separately for males and females) using post hoc Wilcoxon signed-rank tests and corresponding effect sizes calculated using *r*. The group-based mean and standard deviation of VE are provided for the vocally healthy control group as a reference within each sex/loudness condition. Compared with females, the male subgroups had a smaller sample size and did not exhibit the consistent results observed in the female VE subgroup analysis. Figures with box-whisker plots aided in visualizing the significant VE differences exhibited in the female subgroups.

As shown in Table 6 and Figure 4, SPL/Ps demonstrated significant changes following therapy in both low and high subgroups (defined by pretherapy values) in both comfortable and loud conditions. Thus, SPL/Ps may be a reliable indicator of therapeutic outcomes with future work on an expanded data set. The female NPVH subgroups that initially exhibited lower or higher SPL/Ps values migrated toward the normative range observed in vocally healthy individuals after therapy. Following therapy, the migration of SPL/Ps toward the healthy range indicates improved coordination between aerodynamic and acoustic voice subsystems. The significant changes in SPL/Ps in the high and low subgroups highlight the potential of SPL/Ps to reflect changes in vocal function following voice therapy. Traditional VE, SPL/AP, and SPL/AFLOW also demonstrate potential for significant posttherapy changes in the subgroup analysis, although changes were not as evident in the loud condition.

A post hoc ANOVA analysis was run in the female patient group to investigate whether the clinical versus research settings had a significant impact on pre- to posttherapy VE metrics. In particular, the data collection environment in the clinic used a low-bandwidth airflow sensor (PENTAX Corp.) in comparison with a higher bandwidth airflow sensor in the research setting (Glottal Enterprise). Thus, database source was added as a fixed effect. Results confirmed that the three-way interaction among data collection setting, NPVH subgroups, and treatment stage did not have a statistically significant effect on traditional VE, SPL/AP, SPL/Ps, or SPL/AFLOW. Thus, the data collection setting was observed not to have a significant impact on the results.

The posttherapy change in SPL/Ps was not simply due to increases in SPL in the numerator (since loudness is a common target during voice therapy). To determine how therapy was associated with the component parameters of SPL, Ps, and AFLOW, these three parameters were evenly divided into three subgroups—high, middle, and low—based on their pretherapy values. The posttherapy values of each subgroup were then compared with their corresponding pretherapy values (alpha threshold divided by two for statistical significance). Due to violations of normality and homogeneity of variance assumptions, Wilcoxon signed-rank tests were used for these paired comparisons, and effect sizes were calculated using the *r* statistic.

Table 8 summarizes the posttherapy changes in SPL, Ps, and AFLOW for each subgroup, separated by sex and loudness condition. Overall, the treatment-related effects in the low and high subgroups were consistent for females in the comfortable condition. For SPL at comfortable loudness, significant posttherapy changes were exhibited by the low pretherapy SPL subgroup (*p* < .001, *r* = .77) and high pretherapy SPL subgroup (*p* = .019, *r* = −.53). For Ps, posttherapy decreases were observed in the low pretherapy Ps subgroup (*p* = .0071, *r* = .60), middle pretherapy Ps subgroup (*p* = .026, *r* = .52), and high pretherapy Ps subgroup (*p* < .001, *r* = −.71). For AFLOW, a posttherapy decrease was observed in the low pretherapy AFLOW subgroup (*p* = .0078, *r* = .61) and high pretherapy AFLOW subgroup (*p* = .019, *r* = −.55). Notably, in the loud condition, no posttherapy SPL changes were significant. Posttherapy changes were noted for Ps in low and high VE subgroups and AFLOW in the high VE subgroup. This analysis provides evidence that posttherapy changes in VE subgroups are driven by changes in vocal function related to aerodynamic measures of Ps and AFLOW, rather than SPL.

**Table 8.** Sound pressure level (SPL), subglottal pressure (Ps), and airflow (AFLOW) changes in nonphonotraumatic vocal hyperfunction patient subgroups defined by pretherapy categorization.

Subgroup	*N*	SPL (dB SPL)				Ps (cm H_2_O)				AFLOW (L/s)			
		Pre-*M* (*SD*)	Post-*M* (*SD*)	*p*	*r*	Pre-*M* (*SD*)	Post-*M* (*SD*)	*p*	*r*	Pre-*M* (*SD*)	Post-*M* (*SD*)	*p*	*r*
Female, comf													
High	19	84.74 (2.38)	82.14 (4.22)	**.019** *	−.53	11.61 (2.04)	9.34 (2.06)	**< .001** **	−.71	0.25 (0.06)	0.20 (0.08)	**.019** *	−.55
Middle	19	79.75 (1.59)	79.32 (4.70)	.76	−.07	8.06 (0.56)	9.26 (1.99)	**.026** *	.52	0.16 (0.02)	0.17 (0.08)	.81	.06
Low	20	72.00 (3.96)	77.48 (5.77)	**< .001** **	.77	6.27 (0.75)	7.99 (1.89)	**.0071** **	.60	0.09 (0.03)	0.14 (0.06)	**.0078** **	.61
Female, loud													
High	19	91.02 (3.12)	89.09 (4.79)	.083	−.39	16.69 (4.96)	14.03 (3.86)	**.0049** **	−.61	0.23 (0.07)	0.16 (0.08)	**.015** *	−.55
Middle	19	87.33 (4.56)	87.44 (6.80)	.86	.05	13.36 (4.11)	14.38 (3.77)	.49	.17	0.18 (0.05)	0.16 (0.07)	.063	−.43
Low	20	82.60 (5.89)	84.55 (6.69)	.12	.36	11.33 (2.42)	13.23 (3.76)	**.049** *	.45	0.14 (0.06)	0.16 (0.07)	.20	.30
Male, comf													
High	7	85.74 (2.72)	83.00 (6.94)	.46	−.30	11.23 (1.75)	9.93 (2.04)	.15	−.55	0.31 (0.07)	0.26 (0.10)	.11	−.57
Middle	7	79.47 (1.85)	83.60 (3.47)	**.031** *	.83	7.75 (0.78)	8.59 (1.99)	.58	.26	0.21 (0.01)	0.24 (0.07)	.38	.38
Low	8	70.99 (4.00)	81.77 (5.06)	**.016** *	.89	5.51 (1.12)	7.10 (1.45)	.078	.70	0.12 (0.03)	0.13 (0.06)	.93	.06
Male, loud													
High	7	92.73 (2.28)	90.01 (5.93)	.16	−.50	16.88 (7.35)	13.30 (5.48)	.11	−.59	0.32 (0.09)	0.34 (0.12)	.44	.30.
Middle	7	86.90 (2.98)	90.48 (4.69)	.16	.58	14.41 (4.94)	14.67 (6.75)	.94	.06	0.25 (0.12)	0.31 (0.11)	.22	.51
Low	8	78.51 (3.38)	86.81 (7.79)	.11	.64	8.68 (2.80)	10.46 (4.08)	.16	.58	0.14 (0.07)	0.24 (0.11)	.15	.58

*Note.* Bold text indicates statistically significant *p* values. *N* = number of individuals in the group; Pre = pretherapy; Post = posttherapy; comf = comfortable.

* *p* < .05.

** *p* < .01.

### Comparison With SPL/Ps Values in the Literature

Table 9 summarizes the group-wide values for the SPL/Ps ratio that are available in the literature for women with phonotraumatic vocal hyperfunction (Toles et al., 2022) and women and men with unilateral vocal fold paralysis (Bullock et al., 2023). These values are tabulated along with the group-wide measures of SPL/Ps in the current study for vocally healthy women and women with NPVH. As expected, the VE individuals with phonotraumatic vocal hyperfunction and unilateral vocal fold paralysis, as assessed by SPL/Ps, were lower than the VE of vocally healthy speakers. For the patient cohort with NPVH, the average value of SPL/Ps was statistically similar to that of the vocally healthy cohort; this result, as well as the heterogeneity in the presentation of NPVH, motivated the subgroup analysis of the current study.

**Table 9.** Summary of the mean (standard deviation) of sound pressure level over subglottal pressure in vocally healthy females and female individuals with nonphonotraumatic vocal hyperfunction (NPVH; this study), phonotraumatic vocal hyperfunction (PVH; Toles et al., 2022), and unilateral vocal fold paralysis (Bullock et al., 2023).

Loudness condition	Healthy	NPVH	PVH	Paralysis
Comf	9.71 (2.03)	9.69 (2.37)	8.46 (2.17)	9.0 (3.2)
Loud	7.20 (1.61)	6.68 (2.14)	5.91 (1.54)	6.3 (2.5)

*Note.* Comf = comfortable. Units: (dB SPL)/(cm H_2_O).

In terms of treatment-related effects, the SPL/Ps ratio has been shown to detect changes after surgical removal of phonotraumatic vocal fold lesions (Toles et al., 2022). Following laryngeal medialization procedures in the unilateral vocal fold paralysis cohort, SPL/Ps also demonstrated salient treatment-related effects, but only in the patient subgroup exhibiting the largest reductions in AFLOW as a proxy for the largest reductions in glottal gap size (Bullock et al., 2023).

### Limitations

Limitations of this research study warranting future work are described here. Care must be taken when performing post hoc subgrouping of a continuous variable, such as segmenting our patient group into upper, middle, and lower thirds based on VE values. The literature discusses the impact of such artificial grouping that includes attenuation of observed correlations between variables, a reduction in statistical power, and inaccurate estimates (DeCoster et al., 2009, 2011). In certain cases, post hoc subgrouping is warranted to reduce the variability between groups. One such case is an extreme group analysis, such as the one applied in the current work in which the upper and lower thirds of the data set (according to pretherapy metrics) created categorical variables of “high” and “low” VE, respectively. Although the subgrouping is done post hoc, this subgrouping is performed only on the pretherapy data set, which allowed for a statistical analysis of the change in the VE measures within the extreme groups. However, concerns regarding a natural regression to the mean for extreme values temper the interpretation of VE metrics changing solely due to voice therapy.

The sample size was relatively small, with a particular imbalance in terms of male/female distribution. This imbalance might contribute to the large standard deviation observed and the lack of consistent significant changes detected in the male sample. The vocally healthy control group was, on average, 14.2 years older than the patient group. It is possible that age could have affected how normative values of VE were interpreted when compared with the patient cohort; age-related effects could be investigated similar to previous work on SPL, fundamental frequency, and signal-to-noise ratio (Stathopoulos et al., 2011). As a retrospective study, the investigators did not have control over the voice treatment strategies, length of therapy, and length of sessions employed for each client. This disparity in treatment protocols could influence the observed changes in VE ratios. Future studies could aim to explore how different therapy strategies impact VE changes and whether certain approaches yield more favorable outcomes in terms of VE improvements. Finally, the across-gesture variability in mean airflow is known to be variable (Schutte, 1992). Despite this variability across tasks, the importance of airflow should not be neglected. Most voice therapy aims at optimizing airflow to improve VE and voice quality in patients with NPVH (Casper & Murry, 2000; Desjardins et al., 2017; Ramig & Verdolini, 1998).

### Clinical Implications and Future Work

Whereas VE measurement before and after therapy may capture overall treatment effects in patient subgroups, future work could provide additional insight into the dynamics of VE changes over the course of therapy. A more comprehensive assessment approach, with multiple evaluations throughout the treatment process, could help explore the temporal patterns of VE changes. By examining VE during treatment and understanding how they evolve over time, clinicians could be able to better understand the trajectory of therapeutic responses and potentially optimize treatment protocols for each individual. Whereas the current study focused on group-based treatment effects, future work could take a more personalized statistical approach and elucidate vocal function mechanisms in patients who were identified as outlier cases.

Monitoring dynamic VE changes could enable the detection of early stage VE changes deviating from an individual's personal baseline level, which could help in early detection and prevention of NPVH and other voice disorders. In the clinic, the subgrouping of clients with NPVH according to VE could instruct the selection of individualized therapy strategies. Wearable devices are being studied to derive estimates of Ps from neck-surface vibration (Cortés et al., 2022; Marks et al., 2020). Estimates of Ps, along with acoustic measures of voice SPL, could enable individuals and clinicians to track voice use and monitor VE (as measured by SPL/Ps) during daily life. Real-time biofeedback from these devices could aid in developing healthy vocal habits and maintaining vocal hygiene (Van Stan et al., 2022). Further investigations are needed to continue to investigate the role of VE in the assessment, treatment, and prevention of NPVH.

## Conclusions

In conclusion, VE measurements have the potential to complement traditional objective and subjective voice assessments in particular patient cohorts. The four ways of VE calculation demonstrated significant potential as valuable metrics for evaluating the outcome of voice therapy in subsets of individuals with NPVH and aided in stratifying individuals with NPVH into subgroups compared to a vocally healthy group. In particular, SPL/Ps in these subgroups migrated following therapy toward the range observed in healthy controls, which reflects improved coordination between aerodynamic and acoustic voice subsystems. Wearable devices measuring Ps through neck-surface vibration and capturing SPL through microphones could enable individuals and clinicians to track SPL/Ps during daily life, therefore promoting the development of individualized and dynamic therapy based on real-time monitoring of VE metrics.

## Data Availability Statement

Mass General Brigham and Massachusetts General Hospital are not allowed to give access to data without the principal investigator for the human studies protocol first submitting a protocol amendment to request permission to share the data with a specific collaborator on a case-by-case basis. This policy is based on strict rules dealing with the protection of patient data and information. Anyone wishing to request access to the data must contact Sarah DeRosa (sederosa@partners.org), Program Coordinator for Research and Clinical Speech-Language Pathology, Center for Laryngeal Surgery and Voice Rehabilitation, Massachusetts General Hospital.
